# Vimentin neutralizing antibody ameliorate inflammatory bowel disease by inhibiting fibrosis and inflammatory cell death

**DOI:** 10.1371/journal.pone.0352641

**Published:** 2026-10-20

**Authors:** Sol Kim, Hye Yeon Kang, Jin-Sil Park, JeongWon Choi, Ha Yeon Jeong, Seon Yeong Lee, Han Hee Lee, Bo-In Lee, Mi-La Cho

**Affiliations:** 1 Division of Gastroenterology, Department of Internal Medicine, College of Medicine, Seoul St. Mary’s Hospital, The Catholic University of Korea, Seoul, Korea; 2 Lab of Translational ImmunoMedicine, Catholic Research Institute of Medical Science, College of Medicine, The Catholic University of Korea, Seoul, Republic of Korea; 3 Department of Medical Sciences, Graduate School of The Catholic University of Korea, Seoul, Republic of Korea; 4 Department of Pathology, College of Medicine, The Catholic University of Korea, Seoul, South Korea; 5 Mitochondrial Immunomedicine Center for Global Health Therapeutics (MIGHT), Catholic Research Institute of Medical Science, College of Medicine, The Catholic University of Korea, Seocho-gu, Seoul, Republic of Korea; Toho University: Toho Daigaku, JAPAN

## Abstract

**Background:**

Inflammatory bowel disease (IBD) is characterized by chronic inflammation intertwined with fibrosis and increased necroptosis. Vimentin, an intermediate filament protein closely associated with fibrosis and several inflammatory pathways, is elevated in IBD tissues. We conducted experiments to determine the effect of neutralization of extracellular vimentin with an anti-vimentin antibody (Ab) on the pathogenesis of IBD.

**Methods:**

IBD was mimicked in a 7-week C57BL6 mice by mixing 3% dextran sodium sulfate (DSS) in autoclaved drinking water for five days. **A**nti-vimentin Ab was intraperitoneally injected with a concentration of 3 milligrams per kilogram repeatedly in a three-day interval simultaneously with DSS administration.

**Results:**

In dextran sodium sulfate (DSS)-induced colitis mice, the anti-vimentin Ab decreased the levels of total and cell-surface vimentin in colon tissues compared to control-treated mice. In DSS-induced colitis mice, treatment with the anti-vimentin Ab improved body weight loss and colon shortening, thereby reducing the severity of DSS-induced colitis. In addition, the anti-vimentin Ab reduced the number of cells expressing fibrosis markers, inflammatory cytokines, and necroptotic markers in colon tissues of DSS-induced colitis mice. In CCD-18CO human colon epithelial cells, the increases in the levels of fibrosis markers caused by TGFβ were enhanced by vimentin protein stimulation and the anti-vimentin Ab reduced the levels of fibrosis-related factors.

**Conclusion:**

This is the first report of the therapeutic effect of an anti-vimentin Ab on the pathogenic mechanisms of IBD. Further studies are needed to confirm its therapeutic potential in clinical settings.

## Introduction

Inflammatory bowel disease (IBD) is a collection of disease entities characterized by chronic, relapsing, and remitting inflammation within the gastrointestinal tract. IBD comprises Crohn’s disease (CD) and ulcerative colitis (UC). IBD has multiple causes, including genetics, gut alteration of the microbiota, and environmental factors [1]. The disease course typically starts with chronic, aberrant immune response, which triggers extensive tissue damage at the inflammatory site [2]. With an ongoing, unbalanced wound healing response, chronic tissue damage and fibrosis occur, ultimately resulting in organ dysfunction [3].

Vimentin, an intermediate filament protein, regulates key cellular functions such as morphology, adhesion, polarization, stiffness, and migration [4]. Knockout studies in the 1980s revealed that knockout mice (*Vim-/-*) exhibit impaired wound healing, artery remodeling, and vasoactivity due to these cellular defects. Notably, loss of vimentin reduces inflammation and infection, highlighting its role in various human diseases [5–7].

Additional studies revealed that vimentin, both intracellular (iVm) and extracellular (eVm), plays a synergistic role in inflammation. iVm mediates biochemical and mechanical signals from the cell surroundings [5]. It also binds to nucleotide oligomerization domain protein 2 (*NOD2*), activating the nuclear factor kappa-light-chain-enhancer of activated B cells (*NFκB)* pathway and inducing inflammatory cytokines such as IL-1β, IL-6, IL-8, and TNFα and induce autophagy [8,9]. IL-6 then activates Janus kinase (JAK) 1/2—signal transducer and activator of transcription 3 (*STAT3*) axis, further amplifying inflammation. *NOD2*-induced *NFκB* signaling also primes *NLRP3* inflammasome activation through upregulation of *NLRP3* and pro-IL-1β, leading to *Caspase-1*-mediated inflammation and cell death [10].

eVm exists as a soluble or cell surface vimentin (CSV) and is released under cellular stress, inflammation, or cell death [11–13]. Elevated levels of eVim and its modified forms have been observed in inflammatory conditions such as sepsis, pulmonary fibrosis, and rheumatoid arthritis [14–16]. Acting as matrix damage-associated molecular patterns (DAMPs), they bind to pattern recognition receptors (PRRs) such as Toll-like receptor (TLR) 2/4 and integrins, promoting neutrophil migration activating *NFκB* and *STAT3* signaling, which further drives *NLRP3* inflammasome activation [17]. eVm may also indirectly contribute to necroptosis by fostering a pro-inflammatory environment. Necroptosis, mediated by receptor-interacting protein kinase 1/3 (*RIPK1/RIPK3*)-dependent phosphorylation of mixed lineage kinase domain-like protein (*MLKL*), leads to plasma membrane rupture and the release of DAMPs. This process further increases eVim release, creating a vicious inflammatory cycle. Although not directly triggering necroptosis, eVm may promote it by sustaining cytokine-rich conditions [18].

Given these roles, vimentin likely contributes to IBD inflammation. *NOD2* mutations disrupt its interaction with iVm due to alterations in the leucine-rich repeat [19]. This impairs bacterial clearance and dysregulates *NFκB* signaling, leading to chronic inflammation [8,20]. Stress-induced reorganization and extracellular release of vimentin further amplify secondary inflammatory pathways, including inflammasome activation and necroptosis. Studies with *Caspase-8* knockout mice showed that *RIP3* knockdown reduces intestinal inflammation, while *RIP3*-driven necroptosis increases IL-8, IL-1β, and IL-33 levels [21]. Consistently, vimentin expression is elevated in the colonic tissues of CD patients compared to healthy individuals [22].

Vimentin also appears to contribute to IBD-associated fibrosis. Although extracellular matrix deposition is part of normal repair, it becomes excessive in IBD, resulting in submucosal fibrosis and tissue stiffening [23]. Epithelial-mesenchymal transition (EMT), a fibrosis-driving process linked to vimentin, has been implicated in CD pathogenesis [24]. Similarly, UC patients show increased collagen deposition and upregulation of vimentin and other fibrosis markers [25].

Therapeutic targeting of vimentin has shown promise. ALD-R491, a small molecule that binds iVm, significantly reduced colitis severity in dextran sodium sulfate (DSS) models, outperforming tofacitinib and 5-aminosalicylic acid [26]. However, targeting eVm in IBD remains unexplored. Previous studies have used the eVm-targeting monoclonal antibody humanized virus-suppressing factor (hzVSF) to inhibit viral entry in hepatitis B and reduce inflammation in *COVID-19* patients [27,28]. Given eVm’s pro-inflammatory role alongside iVm, we hypothesized that its inhibition could attenuate inflammation and fibrosis. This study aimed to evaluate the therapeutic effects of anti-vimentin antibody treatment on IBD in both *in vivo* and *in vitro* settings.

## Materials and methods

### Animals

Eight-week-old male C57BL/6 mice were purchased from Orient Bio Inc. (Seongnam, Korea). The mice were maintained under specific pathogen-free conditions at the Institute of Medical Science of the Catholic University of Korea with *ad libitum* access to water and were fed standard mouse chow (Ralston Purina, St. Louis, MO, USA). The experimental procedures were performed in accordance with the Laboratory Animals Welfare Act, the Guide for the Care and Use of Laboratory Animals, and the Guidelines and Policies for Rodent Experiments provided by the Department of Laboratory Animals of the Institutional Animal Care and Use Committee of the Catholic University of Korea, and conformed to all the guidelines of the National Institutes of Health (USA) (permit numbers: 2017–0067–02). To minimize animal suffering, mice were fully anesthetized under isoflurane gas (2–2.5%) and were euthanized by cervical dislocation.

### DSS-induced colitis and administration of anti-vimentin Ab

In this study, three groups of mice—normal control, IBD group, and anti-vimentin group, were used to examine the therapeutic implication of anti-vimentin Ab in IBD. Four mouse were allocated to the IBD group, five to the anti-vimentin group, and two to the normal control. IBD was mimicked for both the IBD group and the anti-vimentin Ab group by mixing 3% DSS (MP Biomedicals, Santa Ana, CA, USA) in autoclaved drinking water for five days, whereas the normal control received drinking water without DSS. Anti-vimentin Ab group, unlike the IBD group, received intraperitoneal injection of monoclonal IgG4 anti-vimentin antibody hzVSF-V13 (3 mg/kg; ImmuneMed, Seoul, Korea) in a three-day interval while being fed with DSS mixed drinking water. This antibody is a monoclonal IgG4 antibody that specifically binds to extracellular vimentin. Previous studies have demonstrated its binding specificity, cross-reactivity to both mouse and human vimentin, and therapeutic effects in murine models of viral infection [27,29]. Importantly, hzVSF-v13 has shown cross-reactivity with both human and non-human vimentin, and its IgG4 isotype indicates a non-cytotoxic, immunomodulatory role [30]. This particular concentration was chosen because previous studies have indicated that low to intermediate dose in the range of 1–4 mg/kg yields best clinical results both in animal and human studies [27,30]. The mice were monitored daily for body weight and the presence of blood in the stool. On the eleventh day after DSS administration, mice were euthanized and colons were separated from the small intestine at the ileocecal junction and from the anus at the distal end of rectum. Gross photographs of the colons were taken and their lengths were measured. Colon tissues were fixed in 10% formalin in a cassette for histologic analysis. Data are representative of two independent experiments.

### Assessment of colitis severity

During the DSS treatment cycles, a disease activity index (DAI) score was assessed to evaluate the progression of colitis. The DAI was a composite score determined by factors such as relative body weight loss, stool softness, and blood in the rectum or stool71. Each parameter was scored from 0 to 4, with a maximum cumulative score of 12. Body weight loss was scored as follows: score 0, no weight loss compared to initial weight; 1, weight loss within 1–5%; 2, weight loss within 5–10%; 3, weight loss within 10–20%; 4, greater than 20% weight loss. Stool consistency was determined as follows: score 0, normal (solid pellet); 1, soft but in pellet shape; 2, loose stool but with some solidity; 3, loose stool with signs of liquid consistency; 4, watery diarrhea. Rectal bleeding was scored as follows: score 0, no sign of blood; score 1, no bleeding; 2, slight bleeding; 3, bloody diarrhea; 4, gross bleeding.

Mice that reached a DAI score of 12 were considered to have met the humane endpoint criteria and were euthanized within 24 hours. No animals died before meeting the euthanasia criteria.

### Intracellular staining and flow cytometry

Cells were isolated from the spleen stimulated with 25 ng/mL phorbol myristate acetate and 250 ng/mL ionomycin (Sigma-Aldrich, St. Louis, MO, USA) in the presence of GolgiStop (BD Biosciences, San Jose, CA, USA) for 4 hours. Cells were reacted with anti-CD4 (PerCP Cy5.5, 45-0042-82) and anti-CD25 (APC, 102012) antibodies at 4 °C for 30 minutes, permeabilized with Cytofix/Cytoperm solution (BD Pharmingen, Franklin Lakes, NJ, USA), and stained intracellularly with anti-IL-17 (FITC, 11-7177-81) or anti–Foxp3 (PE, 12-5773-82) antibodies. Samples were analyzed with a FACSCalibur (BD Pharmingen) fluorescence-activated cell sorting (FACS) instrument, and data were analyzed with FlowJo (Tree Star, Ashland, OR, USA).

### Histopathological analysis

Colon tissue sections (5 μm thick) were fixed and embedded in 10% (v/v) neutral-buffered formalin (Sigma-Aldrich, St. Louis, MO). Histological analysis was performed on H&E-stained colitis tissue and scored by two experimenters in a blinded fashion. Stained sections were examined under a photomicroscope (Olympus, Tokyo, Japan) (magnification 200×). The histological scoring system was used for evaluation of the degree of colitis [31]. Loss of epithelium, crypt damage, depletion of goblet cells, and infiltration of inflammatory cells were assessed in the sections. Histological evaluation and scoring of loss of epithelium (0, no loss of epithelium; 1, 0–5% loss of epithelium; 2, 5–10% loss of epithelium; and 3, > 10% loss of epithelium), crypt damage (0, no damage; 1, 0–10% loss of crypt; 2, 10–20% loss of crypt; 3, > 20% loss of crypt), depletion of goblet cells (0, none; 1, mild; 2, moderate; and 3, severe), and infiltration of inflammatory cells (0, none; 1, mild; 2, moderate; and 3, severe) were performed. The total histological score ranged from 0 to 12.

### Immunohistochemistry (IHC)

Formalin-fixed colon sections were paraffin-embedded and 5 μm sections were deparaffinized using xylene and dehydrated in an alcohol series. At least four sections from each colon were analyzed. The sections were treated with proteinase K in tris-ethylenediaminetetraacetic acid buffer for antigen retrieval and washed in 1 × phosphate-buffered saline (PBS; pH 7.5). Endogenous peroxidase activity was quenched with methanol and 3% H_2_O_2_. IHC was performed using the Envision Detection System (Dako, Denmark). The tissues were incubated with primary anti-vimentin (ab92547), anti-CSV (H00007431-M08), anti-IL-17 (PA5–79470), anti-IL-1β (NB600–633), anti-IL-6 (NB600–1131), anti-*TNFα* (ab6671), anti-phosphorylated *STAT3 (pSTAT3*; ab76315), anti-*NFκB* (ab16502), anti-alpha smooth muscle actin (αSMA; ab7817), anti-collagen-1 (MT; PA5–89281), anti-transforming growth factor beta (TGFβ; BS-0086R), anti-*pMLKL* (PA5–105677), anti-*RIP1*, anti-*RIP3* (PA5–19956) and anti-*Caspase-1* (PA5–38099) Abs overnight at 4°C followed by a horseradish peroxidase-conjugated secondary Ab for 30 minutes. The final color product was developed using 3,3-diaminobenzidine (Dako). The sections were counterstained with Mayer’s hematoxylin and examined by photomicroscopy (Olympus).

### Intracellular staining and flow cytometry

Cells were isolated from the spleen, stimulated with 25 ng/mL phorbol myristate acetate and 250 ng/mL ionomycin (Sigma-Aldrich) in the presence of GolgiStop (BD Biosciences, San Jose, CA, USA) for 4 h. Cells were reacted with anti-CD4 (PerCP-Cy5.5, 45-0042-82) and anti-CD25 (APC, 102012) Abs at 4°C for 30 min; permeabilized with Cytofix/Cytoperm solution (BD Pharmingen, Franklin Lakes, NJ, USA); and stained intracellularly with anti-IL-17(FITC, 11-7177-81) or anti-Foxp3 (PE, 12-5773-82) Abs. Samples were analyzed using the Calibur (BD Pharmingen) fluorescence-activated cell sorting (FACS) instrument, and data were analyzed with FlowJo software (Tree Star, Ashland, OR, USA).

### Stimulation of CCD-18CO cells

To confirm the effect of vimentin and anti-vimentin Ab *in vitro*, CCD-18CO cells from Korean Cell Line Bank (Seoul, Korea) were used. The cells were cultured in Dulbecco’s Modified Eagle’s Medium (DMEM, 12,800,017; Gibco, New York, NY, USA), supplemented with 10% (v/v) fetal bovine serum (FBS, #16,000,044; Gibco), and maintained at 37 °C in a 5% CO_2_ atmosphere. CCD-18CO cells were seeded in six-well plates at an initial density of 5 × 10^4^ cells per well and allowed to adhere for 24 hours. Next, the medium was replaced with serum-free DMEM with 1x insulin-transferrin-selenium-sodium pyruvate (ITS-A) solution for 24 hours. The medium was replaced with a fresh medium and the cells were treated with the anti-vimentin Ab (10 μg/mL) or recombinant vimentin (10 ng/mL) with or without 10 ng/mL human recombinant TGFβ for 24 hours. Finally, the cells were harvested for western blotting.

### Protein fractionation

Proteins in the cells were extracted using radioimmunoprecipitation assay (RIPA) lysis and extraction buffer (Cat. 89,901; Thermo Fisher Scientific, Waltham, MA, USA) supplemented with complete protease inhibitor (Cat. 78,438; Thermo) and 0.5 M ethylenediaminetetraacetic acid (EDTA; 78,438; Fisher). After incubating the cocktail solution on ice for 20 minutes, the samples were centrifuged at 14,000 *g* for 15 minutes at 4 °C. The supernatants were collected into new tubes. Protein concentrations were determined using the Pierce BCA Protein Assay Kit (Cat. 23,225, Thermo).

### Immunoblot analysis

Proteins (10 µg) were separated via 10% gradient SDS-PAGE, transferred to Hybond enhanced chemiluminescence (ECL) membranes (10,600,001; Cytiva), and incubated with Abs against anti-alpha smooth muscle actin (α-SMA) (ab7817[1A4]; Abcam, 1:1,000), anti-Col1a1 (PA5–89281; Invitrogen, 1:1,000), anti-fibronectin (ab2414; Abcam, 1:1,000), anti-pSTAT3 (Y705) (#9145; Cell signaling, 1:1000), anti-STAT3 (#9139; Cell signaling, 1:1000), anti-NF-κB p65 (#3033s; Cell signaling, 1:1000), and β-actin (SC-47778; Santa Cruz,:1000) using the SNAP i.d. Protein Detection System (Millipore, Billerica, IL, USA). After incubation with the corresponding secondary anti-mouse (SC-2005; Santa Cruz) and anti-rabbit (SC-2357; Santa Cruz) Abs, protein bands were visualized using X-film (100NIF; EA8EC). Data are representative of two independent experiments.

### Statistical analysis

Statistical analysis was performed using Prism (GraphPad Software, USA). Normally distributed continuous data were analyzed by parametric Student’s *t*-test. Differences in means among groups were subjected to analysis of variance followed by Bonferroni *post hoc* test. Values are means ± standard error of the mean (SEM). Value of *p* < 0.05 was indicative of statistical significance.

### Ethics approval and consent to participate

The animal study was reviewed and approved by the Laboratory Animals Welfare Act (permit number 2024-0183-01], the Guide for the Care and Use of Laboratory Animals, and the Guidelines and Policies for Rodent Experiments of the Department of Laboratory Animals of the Institutional Animal Care and Use Committee of the Catholic University of Korea (permit number 2024-0183-01).

## Results

### The anti-vimentin Ab improved the symptoms of DSS-induced colitis

To investigate the effect of vimentin blockade on the development of IBD, anti-vimentin Ab was intraperitoneally injected repeatedly at 3-day intervals, simultaneously with 3% DSS administration. In DSS-induced colitis mice, the anti-vimentin Ab improved weight loss (Fig 1a). The anti-vimentin Ab also restored colon length on day 11 after treatment, as compared to the control group (6.11 ± 0.12 vs. 5.57 ± 0.06, p < 0.05; Fig 1b). The anti-vimentin Ab treated group had a significantly lower histological score than the DSS group (Fig 1c). Compared to the DSS group, the vimentin Ab-treated group had a smaller fraction of IL-17 + cells (12.14 ± 5.56 vs. 6.67 ± 3.68) and a larger fraction of CD25 + Foxp3 + cells (1.88 ± 0.30 vs. 2.0 ± 0.59) among total *ex vivo* splenic CD4 + cells (Fig 1d). Immunohistochemical analysis was performed on colon tissues to determine the levels of vimentin and CSV. Compared with normal mice, colon tissue sections of DSS-induced colitis mice showed significantly higher total vimentin (21.29 ± 2.37 vs. 10.62 ± 0.87, p < 0.005) and CSV levels (24.52 ± 0.69 vs. 11.08 ± 1.10, p < 0.0001). Treatment with the anti-vimentin Ab reduced the increased total vimentin and CSV levels in colon tissues of DSS-induced colitis mice to levels similar to those in normal mice (Fig 1e).

**Fig 1:**
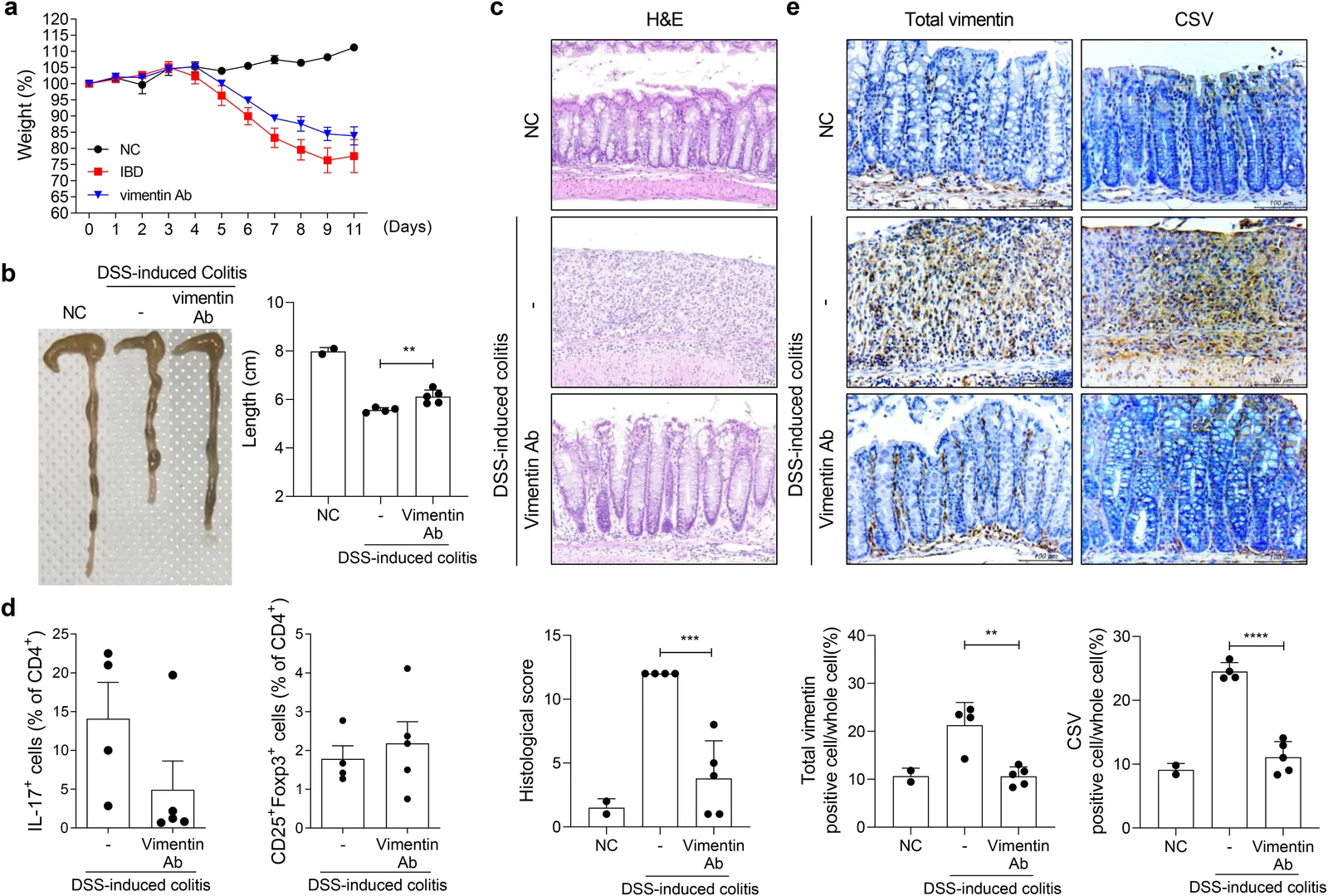
Anti-vimentin Ab improved the symptoms of dextran sulfate sodium-induced ulcerative colitis. Acute colitis was induced in C57BL/6 mice by oral administration of 3% DSS in distilled water for 5 days, followed by regular drinking water for 7 days. Anti-vimentin Ab 3 mg/kg was injected intraperitoneally, simultaneously with DSS injection at 3-day intervals (DSS induced mice, n = 4, Anti-vimentin ab with DSS induced mice, n = 5, normal mice, n = 2). **(a)** Weight expressed as a percentage from day 0. **(b)** Colon length of DSS-induced mice evaluated on day 12 after treatment. **(c)** On day 11, colon tissue sections were stained with hematoxylin and eosin. Representative images are shown (original magnification 200× , scale bar 100 μm). **(d)** Frequencies of IL-17^+^CD4^+^ cells and CD25^+^Foxp3^+^CD4^+^ cells in mouse spleen analyzed by flow cytometry. **(e)** On day 11, colon tissue sections were stained with anti-vimentin and anti-cell surface vimentin Abs. Representative images are shown (original magnification 400 × , scale bar 100 μm). Percentages of positive cells among total cells are shown. Data are means ± SEM (*p < 0.05, **p < 0.005, ****p < 0.001). DSS: Dextran sodium sulfate; Ab: Antibody; NC: Normal control; CSV: Cell surface vimentin; H&E: Hematoxylin and eosin; FACS: Fluorescence-Activated Cell Sorting; Treg: Regulatory T cells.

### The anti-vimentin Ab suppressed inflammation-related transcriptional activity in murine colon tissues with colitis

To determine the effect of the anti-vimentin Ab on the transcriptional activities of pro-inflammatory pathways, *pSTAT3* (Y705) and *NFκB* were examined by immunohistochemical analysis. Compared to normal mice, the colon tissues of DSS-induced colitis mice had larger numbers of *pSTAT3*+ (91.50 ± 6.00 vs. 31.40 ± 5.14, p < 0.0005) and *NFκB*+ (92.25 ± 9.58 vs. 39.80 ± 5.05, p < 0.005) cells. The anti-vimentin Ab significantly reduced the numbers of these cells, as compared to DSS-treated mice (Fig 2a). Confocal immunofluorescence microscopy was performed on colonic tissue sections, staining for CD4, IL-17, CD25, and Foxp3 to identify Th17 and regulatory T (Treg) cells, respectively. Quantification was based on multiple high-power fields per sample. Quantitative analysis revealed that, compared to the DSS group, the anti-vimentin antibody-treated group exhibited fewer IL-17 ⁺ cells (62.75 ± 7.96 vs. 15.40 ± 2.89, p < 0.0005) and a higher number of CD25 ⁺ Foxp3 ⁺ cells (11.50 ± 3.28 vs. 34.80 ± 6.36, p < 0.05) among CD4 ⁺ cells (Fig 2b). These results indicate that blockade of vimentin by an anti-vimentin Ab may influence the transcriptional activities of pro-inflammatory pathways while also modulating intestinal inflammation through interaction with Th17 and Treg cells.

**Fig 2:**
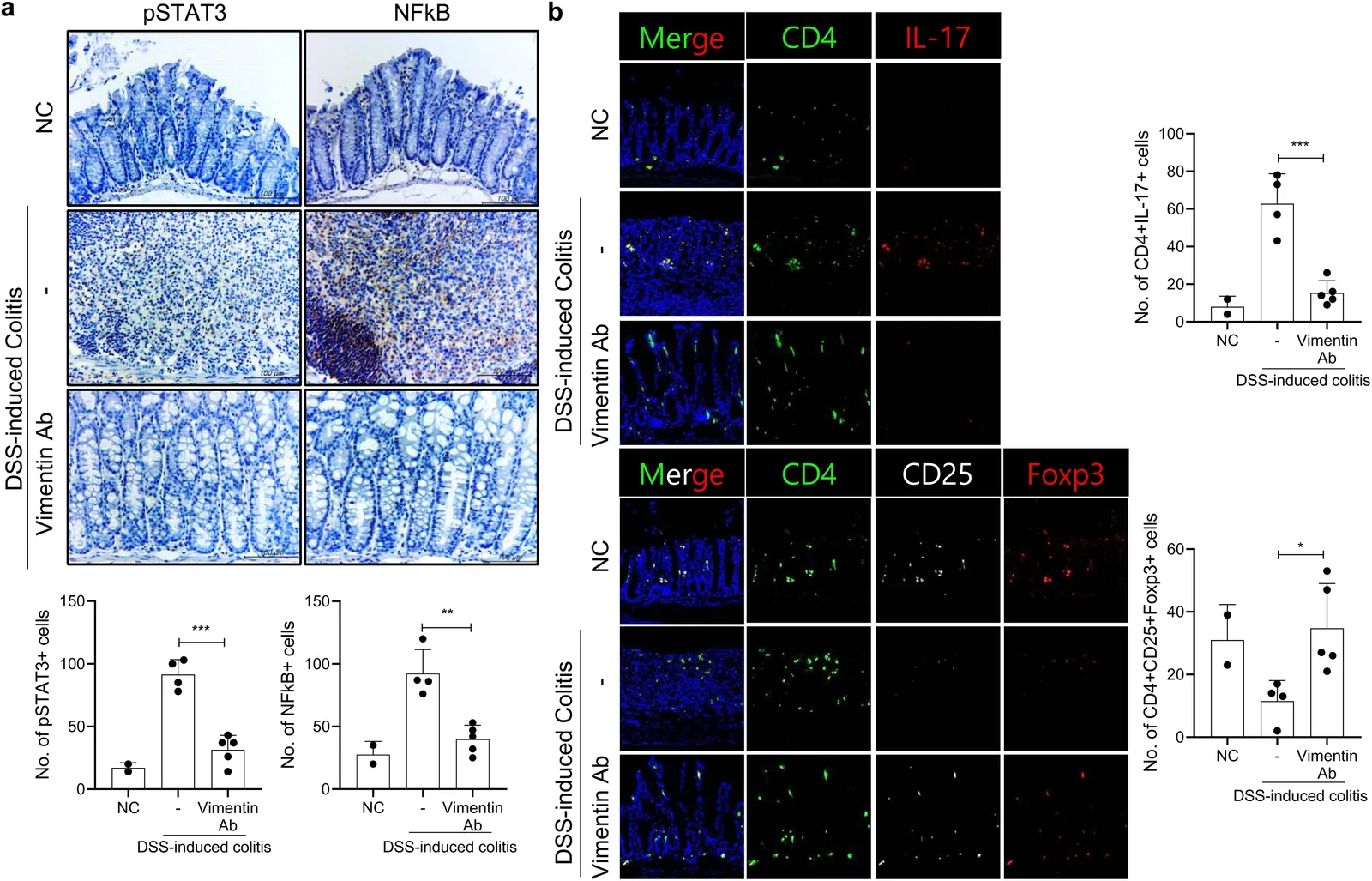
Anti-vimentin Ab suppressed inflammation-related transcriptional activity in DSS-induced colitis mice. Acute colitis was induced in C57BL/6 mice by oral administration of 3% DSS in distilled water for 5 days, followed by regular drinking water for 7 days. Anti-vimentin Ab 3 mg/kg was injected intraperitoneally, simultaneously with DSS injection at 3-day intervals (DSS induced mice, n = 4, Anti-vimentin ab with DSS induced mice, n = 5, normal mice, n = 2). **(a)** On day 11 after DSS administration, colon tissue sections were stained with antibodies for *pSTAT3* and *NFκB*. Representative images are shown (original magnification 400 × , scale bar 100 μm). Percentages of positive cells among total cells are shown. **(b)** Confocal immunofluorescence microscopy was performed on colonic tissue sections, staining for CD4, IL-17, CD25, and Foxp3 to identify Th17 and regulatory T (Treg) cells, respectively. Quantification was based on multiple high-power fields per sample. Data are means ± SEM (*p < 0.05, **p < 0.005, ***p < 0.0005). Ab: Antibody; DSS: Dextran sodium sulfate; IBD: inflammatory bowel disease; No.: Number; NC: Normal control; *pSTAT3*: Phosphorylated signal transducer and activator of transcription 3; *NFκB*: Nuclear factor kappa B; Treg: regulatory T cells.

### The anti-vimentin Ab decreased the number of inflammatory cytokine-secreting cells in murine colon tissue with colitis

Immunohistochemical analysis of colon tissues was performed to determine whether the anti-vimentin Ab reduces the number of inflammatory cytokine-secreting cells in colon tissues of mice with DSS-induced colitis. The anti-vimentin Ab significantly reduced the increased numbers of IL-17 (133.75 ± 15.89 vs. 85.20 ± 7.23, p < 0.05), IL-6 (165.25 ± 16.00 vs. 76.40 ± 5.38, p < 0.0005), *TNFα* (101.00 ± 20.75 vs. 63.60 ± 9.36, p < 0.05), and IL-1β+ (231.50 ± 21.83 vs. 134.80 ± 14.95, p < 0.05) cells in the intestinal tissues of mice with DSS-induced colitis (Fig 3). The results indicate that the anti-vimentin Ab influenced the levels of inflammatory cytokines in inflamed colon tissue.

**Fig 3:**
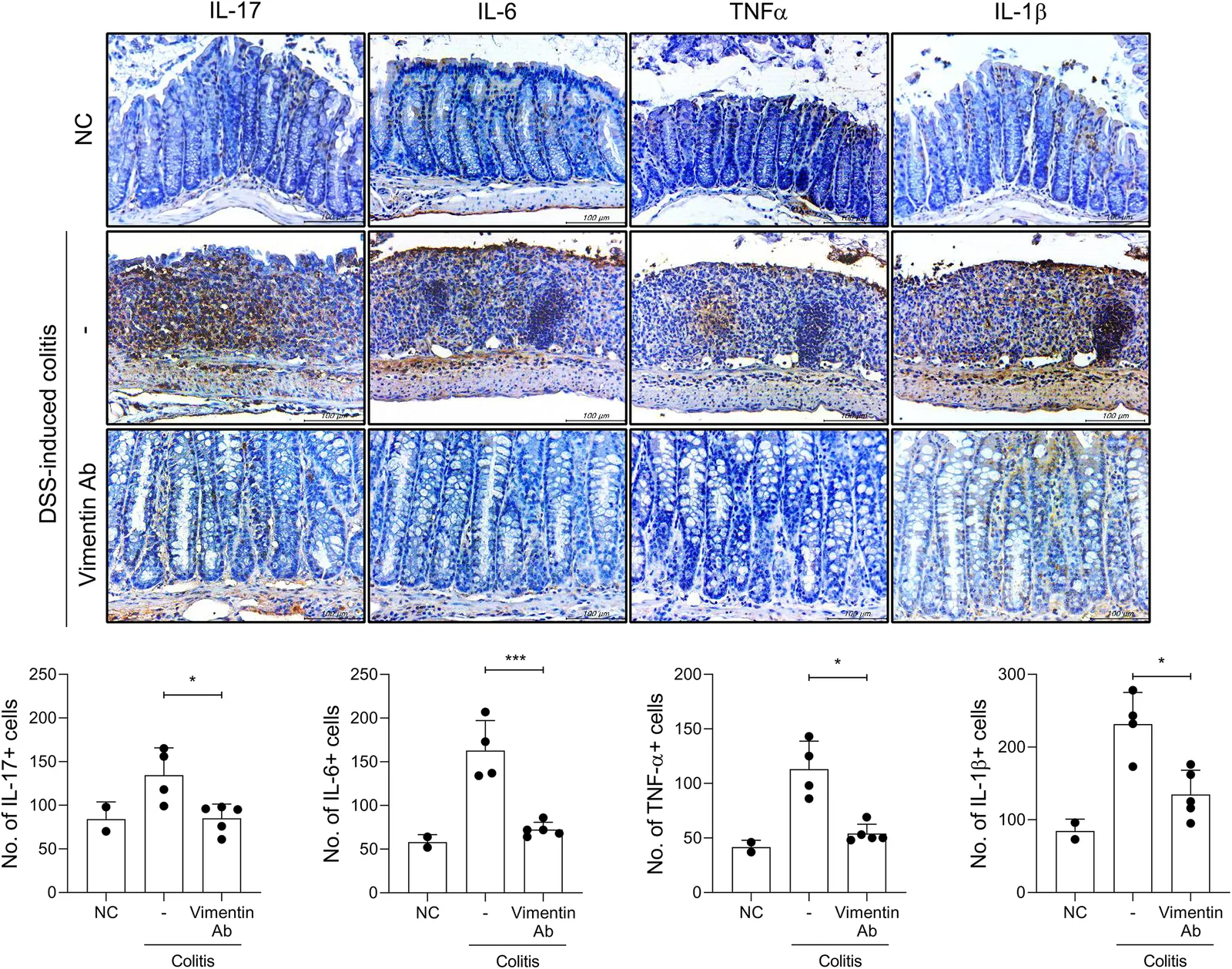
Anti-vimentin Ab decreased the number of inflammatory cells in murine colon tissue with colitis. Acute colitis was induced in C57BL/6 mice by oral administration of 3% DSS in distilled water for 5 days, followed by regular drinking water for 7 days. Anti-vimentin Ab 3 mg/kg was injected intraperitoneally, simultaneously with DSS injection at 3-day intervals (DSS induced mice, n = 4, Anti-vimentin ab with DSS induced mice, n = 5, normal mice, n = 2). On day 11 after DSS administration, colon tissue sections were stained with antibodies for IL-17, IL-6, *TNFα*, and IL-1β. Percentages of positive cells among total cells are shown. Data are means ± SEM (*p < 0.05, ***p < 0.0005). DSS: Dextran sodium sulfate; NC: Normal control; Ab: Antibody; No.: Number; *TNFα*: Tumor necrosis factor alpha.

### The anti-vimentin Ab decreased the levels of necroptosis-related marker in murine colon tissues with colitis

Markers associated with necroptosis- and inflammasome-related inflammatory signaling, including pMLKL, RIPK1, and RIPK3, were detected in colon tissues from mice with DSS-induced colitis. Anti-vimentin Ab treatment was associated with a significant reduction in the numbers of pMLKL⁺ (184.50 ± 11.32 vs. 78.00 ± 4.58, *p* < 0.0001), RIPK1⁺ (253.75 ± 13.31 vs. 139.20 ± 7.51, *p* < 0.0001), RIPK3⁺ (189.50 ± 11.06 vs. 81.00 ± 6.58, *p* < 0.0001), and Caspase-1⁺ (198.75 ± 11.84 vs. 126.80 ± 4.58, *p* < 0.001) cells compared with DSS-treated mice (Fig 4).

**Fig 4:**
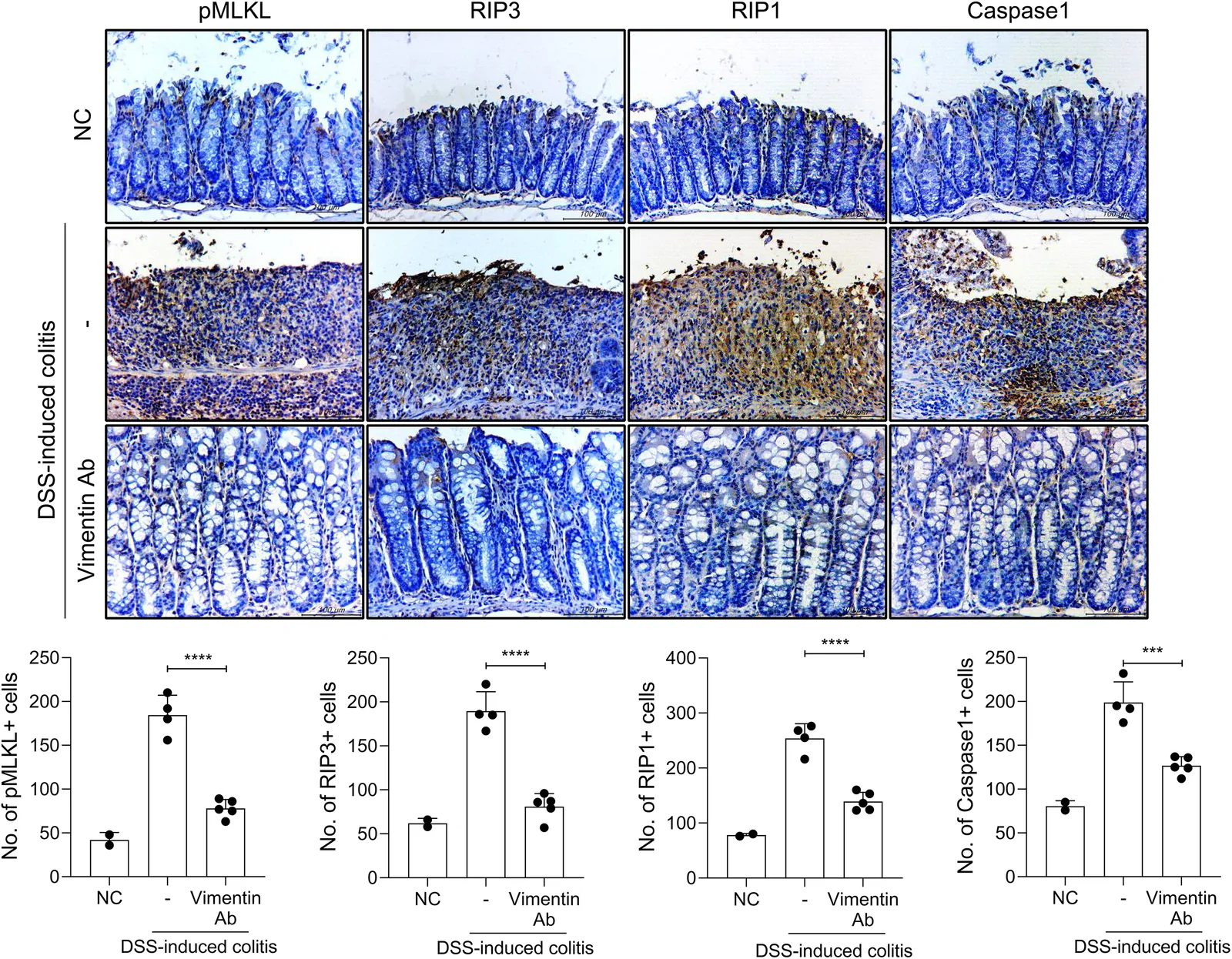
Anti-vimentin Ab decreased the number of inflammatory necroptotic cells in murine colon tissues with colitis. Acute colitis was induced in C57BL/6 mice by oral administration of 3% DSS in distilled water for 5 days, followed by regular drinking water for 7 days. Anti-vimentin Ab 3 mg/kg was injected intraperitoneally, simultaneously with DSS injection at 3-day intervals (DSS induced mice, n = 4, Anti-vimentin ab with DSS induced mice, n = 5, normal mice, n = 2). On day 11 after DSS administration, colon tissue sections were stained with antibodies for *pMLKL*, *RIP3*, *RIP1*, and *Caspase-1*. Percentages of positive cells among total cells are shown. Data are means ± SEM (***p < 0.0005, ****p < 0.001). Ab: Antibody; NC: Normal control; IBD: Inflammatory bowel disease; DSS: Dextran sodium sulfate; No.: Number; *pMLKL*: Phosphorylated mixed lineage kinase domain like pseudokinase; *RIP3*: Receptor-interacting protein kinase-3; *RIP1*: Receptor-interacting protein kinase-1.

These markers are known to be upregulated in inflammatory conditions and are associated with necroptosis- and inflammasome-related signaling pathways. Therefore, the observed reduction following anti-vimentin Ab treatment was interpreted as attenuation of inflammation-associated signaling environments rather than direct inhibition of necroptotic or pyroptotic cell death execution.

### The anti-vimentin Ab reduced the levels of fibrosis-related markers in mice with colitis

To investigate the antifibrotic effect of the anti-vimentin Ab in mice with DSS-induced colitis, colon sections were stained with Masson’s trichrome. In mice with colitis, increased collagen deposition and severe intestinal structure damage due to infiltration of inflammatory cells were observed. These effects were in part reversed by the anti-vimentin Ab. Compared to normal mice, the colon tissue of mice with DSS-induced colitis had larger numbers of cells expressing fibrosis-related markers in αSMA (153.00 ± 9.57 vs. 98.80 ± 7.81, p < 0.005), Col1α1 (165.25 ± 16.00 vs. 74.20 ± 7.39, p < 0.0005), and TGFβ (101.00 ± 20.75 vs. 69.4 ± 12.11). The anti-vimentin Ab significantly reduced the numbers of cells expressing fibrosis-related markers, compared to DSS-treated mice (Fig 5).

**Fig 5:**
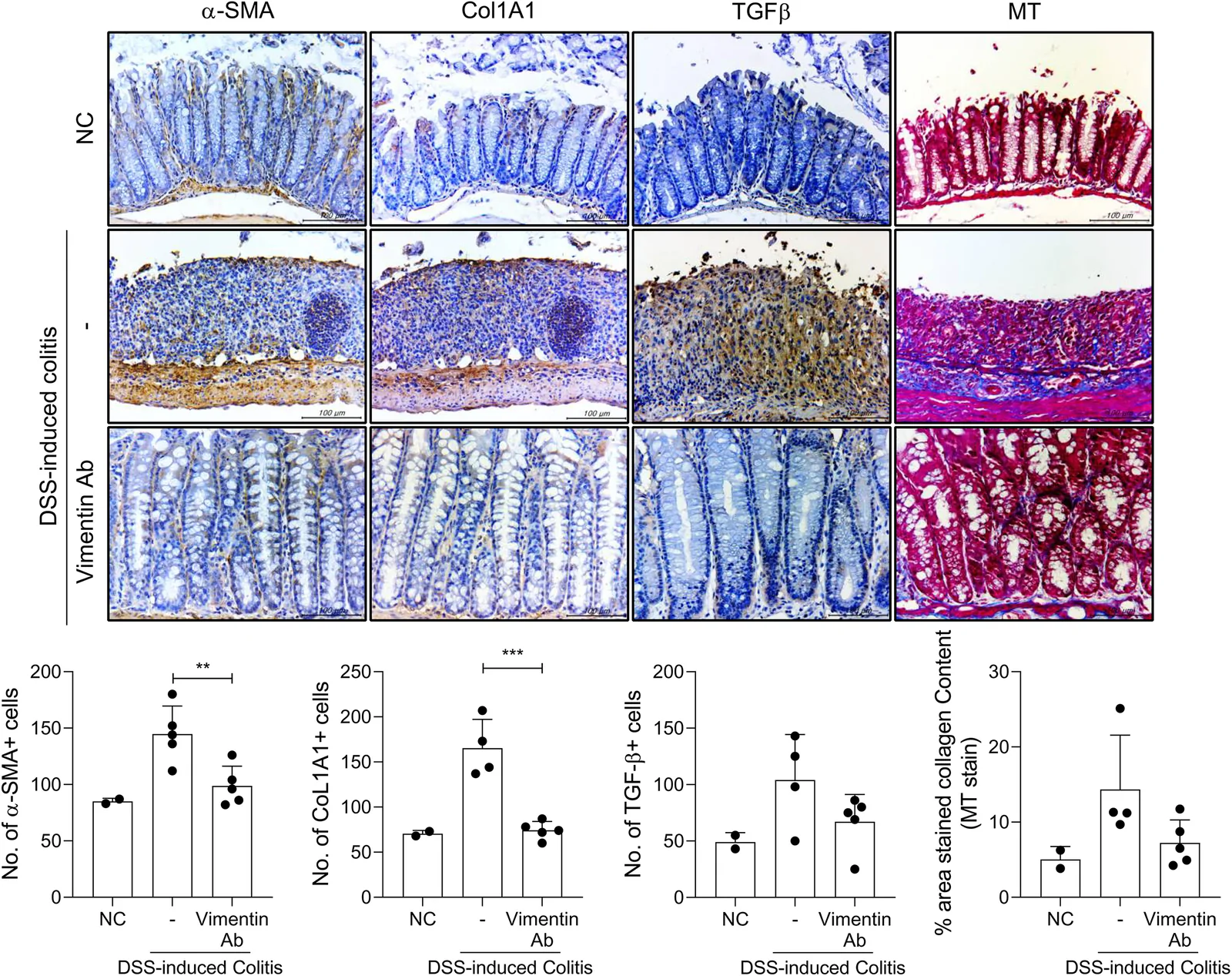
Anti-vimentin Ab reduced fibrosis in mice with colitis. Acute colitis was induced in C57BL/6 mice by oral administration of 3% DSS in distilled water for 5 days, followed by regular drinking water for 7 days. Anti-vimentin Ab 3 mg/kg was injected intraperitoneally, simultaneously with DSS injection at 3-day intervals (DSS induced mice, n = 4, Anti-vimentin ab with DSS induced mice, n = 5, normal mice, n = 2). **(a)** On day 11 after DSS administration, colon sections were stained with Masson’s trichrome. The percentage collagen-positive area was analyzed as ImageJ. **(b)** Colon tissue sections were stained with antibodies for α-SMA, Col1A1, and TGFβ. Numbers of antibody-positive cells are shown. Data are means ± SEM (**p < 0.005, ***p < 0.0005). DSS: dextran sodium sulfate; Ab: Antibody; NC: Normal control; IBD: Inflammatory bowel disease; αSMA: Alpha smooth muscle actin; Col1α1: Collagen type I alpha 1; TGFβ: Transforming growth factor beta; MT: Masson’s trichrome stain for collagen.

### The anti-vimentin Ab reduced fibrosis-related response and increased inflammation in human colon epithelial cells *in vitro*

We investigated the effect of vimentin on fibrosis using CCD-18CO human colon epithelial cells. When cells were treated with TGF-β and cultured for 24 hours, the levels of fibrosis-related factors such as α-SMA (1.75 ± 0.17 vs. 1.07 ± 0.20), Col1a1 (1.20 ± 0.49 vs. 2.30 ± 0.37), and fibronectin (3.24 ± 1.22 vs. 2.30 ± 0.37) increased, with the change in fibronectin showing statistical significance. Treatment with vimentin further increased the levels of fibrosis-related factors induced by TGF-β (Fig 6a). To determine whether blocking vimentin improves fibrosis in human colon epithelial cells, TGF-β and the anti-vimentin antibody were added to CCD-18CO cells. In cells concomitantly administered the anti-vimentin antibody, the levels of fibrosis markers decreased, and the reduction in fibronectin expression was statistically significant (α-SMA: 1.21 ± 0.49 vs. 1.76 ± 0.64, Col1α1: 1.20 ± 0.49 vs. 2.30 ± 0.37, fibronectin 1.61 ± 0.51 vs. 3.34 ± 0.49; Fig 6b). Lastly, the effect of anti-vimentin antibody treatment on the transcriptional activity of pro-inflammatory pathways—pSTAT3 and NF-κB—was evaluated by western blot analysis. Upon treatment with TGF-β for 24 hours, both pSTAT3 and NF-κB protein levels were elevated. The addition of anti-vimentin antibody significantly reduced the expression of pSTAT3 (1.57 ± 0.18 vs. 1.12 ± 0.28, p < 0.05) and NF-κB (1.56 ± 0.11 vs. 1.00 ± 0.18, p < 0.05) protein levels (Fig 6c).

**Fig 6:**
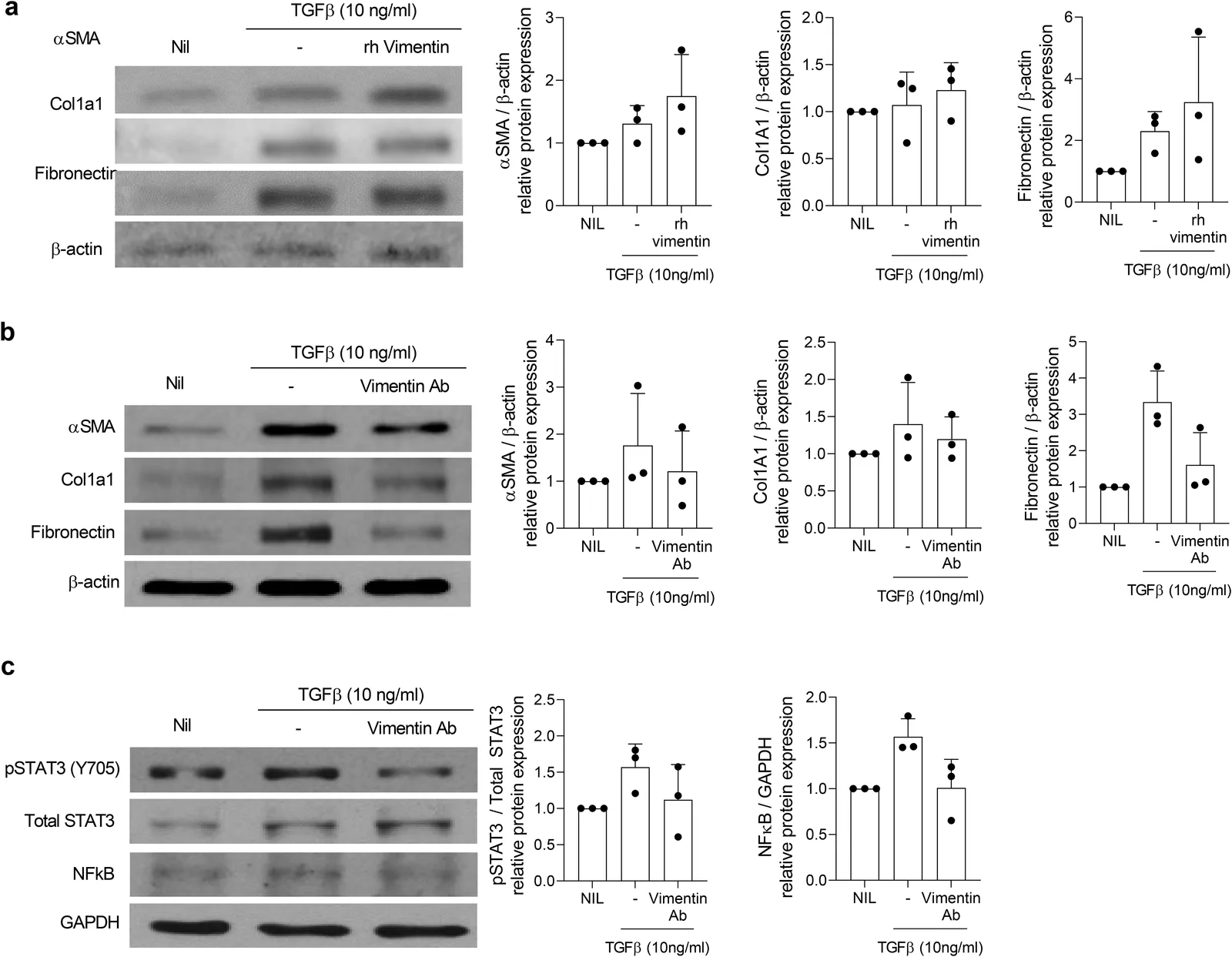
The anti-vimentin Ab reduced fibrosis and increased inflammation in human colon epithelial cells *in vitro.* CCD-18CO cells were starved for 24 hours with serum-free DMEM and stimulated with recombinant vimentin (10 ng/mL) (a) or anti-vimentin Ab (10 μg/mL) (b) with or without TGFβ (10 ng/mL) for 24 hours. The levels of αSMA, Col1a1, and fibronectin were analyzed by western blotting. (c) the effect of anti-vimentin antibody treatment on the transcriptional activity of pro-inflammatory pathways—*pSTAT3* (Y705) and *NFκB*—was evaluated by western blot analysis. Data are means ± SEM (*p < 0.05**p < 0.005). Ab: Antibody; αSMA: Alpha smooth muscle actin; Col1α1: Collagen type I alpha 1; TGFβ: Transforming growth factor beta; ng: Nanogram; ml: Milliliter; rh: Recombinant human; *pSTAT3*: Phosphorylated signal transducer and activator of transcription 3; *NFκB*: Nuclear factor kappa **B.**

## Discussion

Vimentin is a potential therapeutic target in IBD, as indicated by its upregulation following tissue injury and its involvement in multiple signaling pathways linked to cell migration during wound repair. It also modulates the functions of immunocytes and participates in the pro-inflammatory responses required to fend off infection by interacting with inflammasome *NLRP3* and inducing cytokine production [32]. This process is also in part mediated by necroptosis, which is a result of continued mucosal barrier disruption in IBD. We aimed to recreate the inflammatory environment of IBD in mouse and determine the effect of controlling the eVm level using an anti-vimentin Ab.

DSS-induced colitis mice displayed clinical characteristics that resembled IBD. The overall weight, length, and histological score of colon samples were severely impaired, possibly due to extensive colonic inflammation. The mice treated with the anti-vimentin Ab showed better clinical outcomes than those with DSS-induced colitis (Fig 1a, b, c). In *ex vivo* spleen FACS, the anti-vimentin Ab-treated mice with DSS-induced colitis had a lower percentage of cells with IL-17 and a higher percentage of Treg cells (Fig 1d). Likewise, confocal immunofluorescence microscopy on colonic tissue sections revealed that compared to the DSS group, the anti-vimentin antibody-treated group exhibited fewer IL-17 ⁺ cells and a higher number of CD25 ⁺ Foxp3 ⁺ cells among CD4 ⁺ cells (Fig 2b). Based on the roles of IL-17 and Tregs in innate immunity, it can be inferred that inhibiting eVim can modulate intestinal inflammation. This correlation was confirmed by immunochemical analysis of the vimentin levels in normal mice, mice with DSS-induced colitis, and anti-vimentin Ab-treated mice with DSS-induced colitis. Colon tissues from the DSS-induced colitis group had the highest total vimentin and CSV; tissue in the vimentin Ab treated DSS-induced colitis group was similar to that of normal mice (Fig 1e). Intraperitoneal injection of the anti-vimentin Ab to mice treated with different DSS concentrations resulted in similar weights, colon lengths, and histologic scores.

Our findings implicate vimentin in multiple pathogenic mechanisms of IBD, namely, inflammation, fibrosis-related response, and necroptosis. Both *in vivo* and *in vitro* studies showed that the levels of fibrosis-related response markers were elevated in the mice with DSS-induced colitis, as compared to anti-vimentin Ab treated mice with DSS-induced colitis (Figs 5, 6a, 6b). This is in agreement with reports of a link between increased collagen deposition and fibrosis with upregulation of vimentin. Given the role of vimentin intermediate filaments in wound healing and fibrosis, this is unsurprising. This dependency of fibrosis on the eVm level suggests that the severity of fibrosis in IBD can be ameliorated by an anti-vimentin Ab that targets eVim including CSV. Although differences in the expression of fibrosis-associated markers such as αSMA, Col1a1, and fibronectin between the DSS group and the anti-vimentin Ab group were not statistically significant in an *in vitro* study using CCD-18CO human colon cell line, we believe this may be attributed to the limited number of replicates rather than a lack of biological effect. This is further supported by the significant anti-fibrotic effects observed in our *in vivo* murine models. Follow-up studies with larger sample sizes and optimized in vitro conditions to further assess the therapeutic potential of anti-vimentin antibody in human colon-derived cells is needed.

Controlling the vimentin level suppressed several pro-inflammatory pathways and inflammatory cytokine production. The activation levels of pro-inflammatory pathways in *pSTAT3* and *NFκB* were higher in the mice with DSS-induced colitis than in the anti-vimentin Ab-treated mice with DSS-induced colitis (Fig 2a). Similar results were obtained for the inflammatory cytokines IL-17, IL-6, *TNFα*, and IL-1β (Fig 3). The DSS-induced colitis group had higher levels of these cytokines than the anti-vimentin Ab-treated DSS-induced colitis group. *In vitro* western blot analysis of colon tissue lysates revealed a similar pattern. Upon treatment with TGFβ, both *pSTAT3* and *NFκB* protein levels were elevated. The addition of anti-vimentin antibody appeared to reduce the expression of both proteins; however, these reductions did not reach statistical significance, which we believe has to do with the limited number of replicates rather than an absence of biological effect (Fig 6c). The fact that *in vitro* study displayed a similar trend in pSTAT3 and NFκB upon administration of anti-vimentin Ab is nonetheless a noteworthy finding. Considering eVm’s ability to activate *NFκB*/*STAT3* pathway and further trigger *NLRP3* inflammasome activation, our results are in accordance with previous findings and demonstrate the importance of vimentin in the inflammatory pathogenesis of IBD [8,9].

Cell necroptosis is a key driver of inflammation initiation and aggravation [33]. Damage to the intestinal mucosal barrier, and the subsequent higher permeability of intestinal epithelial cells, may contribute to the development of IBD [34]. Necroptosis, a process of cell death that is normally regulated, is implicated in the development of intestinal inflammation [20]. Studies using *caspase-8* knockout mice have shown that RIP3 knockdown ameliorates intestinal inflammation, whereas necroptosis induced by *RIP3* increased the expression of the inflammatory cytokines IL-8, IL-1β, and IL-33, among others [21]. Necroptosis induces translocation of *NFKβ* and assembly of the *NLRP3* inflammasome [35]. These mouse studies have shown that necroptosis of intestinal epithelial cells aggravates intestinal inflammation. Regulation of necroptosis can suppress UC, but the serum level of *TNFα* and the mucosal tissue level of *RIP3* are increased in patients with CD [20]. Although this study was not aimed at suppressing necroptosis, we found that regulating eVm with the anti-vimentin Ab suppressed the levels of necroptotic markers. The anti-vimentin Ab-treated DSS-induced colitis group showed decreased levels of necroptotic markers (*pMLKL, RIP3, RIP1,* and *Caspase1*), as compared to the DSS-induced colitis group (Fig 5). While eVm does not directly exert influence on neroptosis, blocking eVm indirectly attenuate necroptosis by reducing inflammation, lowering *TNFα* levels, and disrupting DAMP-induced signaling loops [36].

A limitation of this study is the lack of analysis on the dose-dependent effects of anti-vimentin antibody treatment. We used a single dosage of 3 mg/kg, based on its demonstrated efficacy in prior studies. In a woodchuck hepatitis virus model, Korolowicz et al. (2021) tested multiple doses (0.1, 4.0, and 16.0 mg/kg) and found that an intermediate dose yielded the strongest therapeutic response. Similarly, in a clinical trial for COVID-19, investigators reported better outcomes with a lower dose (200 mg) compared to a higher dose (400 mg) (Kang, Choe et al. 2020, Prasenohadi, Burhan et al. 2022). In our preliminary tests, a higher dose (9 mg/kg) produced comparable therapeutic outcomes but was associated with increased mortality. Based on these findings, we proceeded with the 3 mg/kg dosage. Future studies are needed to determine whether anti-vimentin antibody efficacy is dose-dependent within a lower dose range.

In summary, an Ab that reduced the extracellular vimentin level improved the clinical symptoms of IBD in a mouse model. These improved symptoms were correlated with decreased fibrosis, inflammation, and necroptosis *in vivo* and *in vitro*. To our knowledge, this is the first attempt to use an Ab targeting vimentin in IBD. Currently, IBD requires multimodal therapy that includes a wide spectrum of medications from aminosalicylates to biologics such as anti-TNF agents or anti-IL-23 antibodies. Encompassing anti-vimentin Ab, which offers a symptomatic aid by effecting a novel pathway, as a part of this multimodal approach would provide a more comprehensive method of attacking IBD pathophysiology. Further studies should analyze whether the effect is dose dependent, and whether the results would be replicated in human.

## References

[1] Zhou M, He J, Shen Y, Zhang C, Wang J, Chen Y. New frontiers in genetics, gut microbiota, and immunity: a rosetta stone for the pathogenesis of inflammatory bowel disease. Biomed Res Int. 2017;2017:8201672. 10.1155/2017/8201672 PMC5558637 28831399

[2] McGuckin MA, Eri R, Simms LA, Florin THJ, Radford-Smith G. Intestinal barrier dysfunction in inflammatory bowel diseases. Inflamm Bowel Dis. 2009;15(1):100–13. doi: 10.1002/ibd.20539 18623167

[3] Wynn TA, Ramalingam TR. Mechanisms of fibrosis: therapeutic translation for fibrotic disease. Nat Med. 2012;18(7):1028–40. doi: 10.1038/nm.2807 22772564 PMC3405917

[4] Ridge KM, Eriksson JE, Pekny M, Goldman RD. Roles of vimentin in health and disease. Genes Dev. 2022;36(7–8):391–407. doi: 10.1101/gad.349358.122 35487686 PMC9067405

[5] Danielsson F, Peterson MK, Caldeira Araújo H, Lautenschläger F, Gad AKB. Vimentin Diversity in Health and Disease. Cells. 2018;7(10). 10.3390/cells7100147 PMC6210396 30248895

[6] Langlois B, Belozertseva E, Parlakian A, Bourhim M, Gao-Li J, Blanc J, et al. Vimentin knockout results in increased expression of sub-endothelial basement membrane components and carotid stiffness in mice. Sci Rep. 2017;7(1):11628. doi: 10.1038/s41598-017-12024-z 28912461 PMC5599644

[7] Paulin D, Lilienbaum A, Kardjian S, Agbulut O, Li Z. Vimentin: Regulation and pathogenesis. Biochimie. 2022;197:96–112. 10.1016/j.biochi.2022.02.003 35151830

[8] Stevens C, Henderson P, Nimmo ER, Soares DC, Dogan B, Simpson KW, et al. The intermediate filament protein, vimentin, is a regulator of NOD2 activity. Gut. 2013;62(5):695–707. doi: 10.1136/gutjnl-2011-301775 22684479 PMC4225453

[9] Henderson P, Wilson DC, Satsangi J, Stevens C. A role for vimentin in Crohn disease. Autophagy. 2012;8(11):1695–6. doi: 10.4161/auto.21690 22929019 PMC3494605

[10] Zheng D, Liwinski T, Elinav E. Inflammasome activation and regulation: toward a better understanding of complex mechanisms. Cell Discov. 2020;6:36. doi: 10.1038/s41421-020-0167-x 32550001 PMC7280307

[11] Walker JL, Bleaken BM, Romisher AR, Alnwibit AA, Menko AS. In wound repair vimentin mediates the transition of mesenchymal leader cells to a myofibroblast phenotype. Mol Biol Cell. 2018;29(13):1555–70. doi: 10.1091/mbc.E17-06-0364 29718762 PMC6080657

[12] Sadeghi N, Fazli G, Bayat AA, Fatemi R, Ebrahimnejhad N, Salimi A, et al. Cell Surface Vimentin Detection in Cancer Cells by Peptide-Based Monoclonal Antibody. Avicenna J Med Biotechnol. 2023;15(2):68–75. doi: 10.18502/ajmb.v15i2.12016 37034891 PMC10073919

[13] Amraei R, Xia C, Olejnik J, White MR, Napoleon MA, Lotfollahzadeh S, et al. Extracellular vimentin is an attachment factor that facilitates SARS-CoV-2 entry into human endothelial cells. Proc Natl Acad Sci U S A. 2022;119(6):e2113874119. doi: 10.1073/pnas.2113874119 35078919 PMC8833221

[14] Van Steendam K, Tilleman K, Deforce D. The relevance of citrullinated vimentin in the production of antibodies against citrullinated proteins and the pathogenesis of rheumatoid arthritis. Rheumatology (Oxford). 2011;50(5):830–7. doi: 10.1093/rheumatology/keq419 21278075 PMC3077912

[15] Miao H, Cui Y, Lu Y, Sun T, Dou J, Ren Y, et al. Serum vimentin predicts mortality in pediatric severe sepsis: A prospective observational study. Int J Infect Dis. 2022;121:141–7. doi: 10.1016/j.ijid.2022.05.018 35568360

[16] Li FJ, Surolia R, Li H, Wang Z, Liu G, Kulkarni T, et al. Citrullinated vimentin mediates development and progression of lung fibrosis. Sci Transl Med. 2021;13(585):eaba2927. doi: 10.1126/scitranslmed.aba2927 33731433 PMC8594069

[17] Suprewicz Ł, Fiedoruk K, Skłodowski K, Hutt E, Zakrzewska M, Walewska A, et al. Extracellular vimentin is a damage-associated molecular pattern protein serving as an agonist of TLR4 in human neutrophils. Cell Commun Signal. 2025;23(1):64. doi: 10.1186/s12964-025-02062-w 39910535 PMC11800445

[18] Zhou P, Zhang S, Wang M, Zhou J. The Induction Mechanism of Ferroptosis, Necroptosis, and Pyroptosis in Inflammatory Bowel Disease, Colorectal Cancer, and Intestinal Injury. Biomolecules. 2023;13(5):820. doi: 10.3390/biom13050820 37238692 PMC10216135

[19] Eckmann L, Karin M. NOD2 and Crohn’s disease: loss or gain of function? Immunity. 2005;22(6):661–7. doi: 10.1016/j.immuni.2005.06.004 15963781

[20] Li S, Ning L-G, Lou X-H, Xu G-Q. Necroptosis in inflammatory bowel disease and other intestinal diseases. World J Clin Cases. 2018;6(14):745–52. doi: 10.12998/wjcc.v6.i14.745 30510938 PMC6265005

[21] Günther C, Martini E, Wittkopf N, Amann K, Weigmann B, Neumann H, et al. Caspase-8 regulates TNF-α-induced epithelial necroptosis and terminal ileitis. Nature. 2011;477(7364):335–9. doi: 10.1038/nature10400 21921917 PMC3373730

[22] Osman NH, Jailani RF, Rahman HA, Hamid NA. Gene and protein expression of epithelial to mesenchymal transition for intestinal and anal fistula: a systematic review. Ann Coloproctol. 2023;39(2):106–14. doi: 10.3393/ac.2021.00584.0083 34856655 PMC10169555

[23] Rieder F, Mukherjee PK, Massey WJ, Wang Y, Fiocchi C. Fibrosis in IBD: from pathogenesis to therapeutic targets. Gut. 2024;73(5):854–66. doi: 10.1136/gutjnl-2023-329963 38233198 PMC10997492

[24] Jiang H, Shen J, Ran Z. Epithelial-mesenchymal transition in Crohn’s disease. Mucosal Immunol. 2018;11(2):294–303. doi: 10.1038/mi.2017.107 29346350

[25] Ippolito C, Colucci R, Segnani C, Errede M, Girolamo F, Virgintino D, et al. Fibrotic and Vascular Remodelling of Colonic Wall in Patients with Active Ulcerative Colitis. J Crohns Colitis. 2016;10(10):1194–204. doi: 10.1093/ecco-jcc/jjw076 26995183

[26] Wu J, Wu X, Cheng C, Liu L, Xu L, Xu Z, et al. Therapeutic targeting of vimentin by ALD-R491 impacts multiple pathogenic processes to attenuate acute and chronic colitis in mice. Biomed Pharmacother. 2023;168:115648. doi: 10.1016/j.biopha.2023.115648 37812892

[27] Prasenohadi P, Burhan E, Dhunny S, Suharno W, Wabnitz P, Kim Y-W, et al. Double-Blind, Randomized, Placebo-Controlled Study on hzVSF-v13, a Novel Anti-Vimentin Monoclonal Antibody Drug as Add-on Standard of Care in the Management of Patients with Moderate to Severe COVID-19. J Clin Med. 2022;11(11):2961. doi: 10.3390/jcm11112961 35683351 PMC9181020

[28] Kang CK, Choe PG, Park S, Kim TS, Seong M-W, Kim N-J, et al. Compassionate use of hzVSF-v13 in two patients with severe COVID-19. J Med Virol. 2020;92(11):2371–3. doi: 10.1002/jmv.26063 32458425 PMC7283658

[29] Korolowicz KE, Suresh M, Li B, Huang X, Yon C, Kallakury BV, et al. Combination Treatment with the Vimentin-Targeting Antibody hzVSF and Tenofovir Suppresses Woodchuck Hepatitis Virus Infection in Woodchucks. Cells. 2021;10(9):2321. doi: 10.3390/cells10092321 34571970 PMC8466705

[30] Miyakawa Y, Otsuka M, Sekiba K, Funato K, Koike K. Humanized virus-suppressing factor inhibits hepatitis B virus infection by targeting viral cell entry. Heliyon. 2021;7(7):e07586. doi: 10.1016/j.heliyon.2021.e07586 34345745 PMC8319007

[31] Han ES, Oh JY, Park H-J. Cordyceps militaris extract suppresses dextran sodium sulfate-induced acute colitis in mice and production of inflammatory mediators from macrophages and mast cells. J Ethnopharmacol. 2011;134(3):703–10. doi: 10.1016/j.jep.2011.01.022 21277968

[32] Wang L, Mohanasundaram P, Lindström M, Asghar MN, Sultana G, Misiorek JO, et al. Vimentin Suppresses Inflammation and Tumorigenesis in the Mouse Intestine. Front Cell Dev Biol. 2022;10:862237. doi: 10.3389/fcell.2022.862237 35399505 PMC8993042

[33] Duan C, Xu X, Lu X, Wang L, Lu Z. RIP3 knockdown inhibits necroptosis of human intestinal epithelial cells via TLR4/MyD88/NF-κB signaling and ameliorates murine colitis. BMC Gastroenterol. 2022;22(1):137. doi: 10.1186/s12876-022-02208-x 35346043 PMC8961930

[34] Jergens AE, Parvinroo S, Kopper J, Wannemuehler MJ. Rules of Engagement: Epithelial-Microbe Interactions and Inflammatory Bowel Disease. Front Med (Lausanne). 2021;8:669913. doi: 10.3389/fmed.2021.669913 34513862 PMC8432614

[35] Negroni A, Colantoni E, Pierdomenico M, Palone F, Costanzo M, Oliva S, et al. RIP3 AND pMLKL promote necroptosis-induced inflammation and alter membrane permeability in intestinal epithelial cells. Dig Liver Dis. 2017;49(11):1201–10. doi: 10.1016/j.dld.2017.08.017 28844856

[36] Thalla DG, Jung P, Bischoff M, Lautenschläger F. Role of Extracellular Vimentin in Cancer-Cell Functionality and Its Influence on Cell Monolayer Permeability Changes Induced by SARS-CoV-2 Receptor Binding Domain. Int J Mol Sci. 2021;22(14):7469. doi: 10.3390/ijms22147469 34299089 PMC8303762

